# Pediatric Hydatidosis in Iranian Referral Pediatrics Center

**Published:** 2012

**Authors:** S Mahmoudi, S Elikaee, H Keshavarz, B Pourakbari, S Mamishi

**Affiliations:** 1 Pediatric Infectious Diseases Research Center, Tehran University of Medical Sciences, Tehran, Iran; 2 Department of Medical Parasitology and Mycology, School of Public Health, Tehran University of Medical Sciences, Tehran, Iran; 3 Center for Research of Endemic Parasites of Iran (CREPI), Tehran University of Medical Sciences, Tehran, Iran; 4 Department of Infectious Disease, School of Medicine, Tehran University of Medical Sciences, Tehran, Iran

**Keywords:** Hydatid cyst, Clinical manifestations, Children, Iran

## Abstract

**Background:**

Hydatidosis is one of the major zoonotic diseases that cause considerable public health problems in Iran. The present study was designed to investigate pediatric hydatidosis in patients referred to the Children Medical Center Hospital in Tehran, Iran during 2005-2010.

**Methods:**

Data were collected from the records of 17 patients referred to the center with hydatidosis. Data included demographic data; laboratory results, type, and site of cysts, clinical manifestations, and treatment.

**Results:**

Nine patients were boys (52.9%) and eight (47.1%) were girls. Most patients referred from central areas of Iran (58.8%). Seven patients had cysts in their lungs (41.2%) and three cases (17.6%) in liver. Six cases (35.3%) had simultaneous lung and liver cysts, 3 patients (17.6%) had brain cysts (alone or in combination with other organs involvement) and 2 patients (11.7%) showed multi-organ involvement. All patients were treated by albendazole and underwent surgery, recurrence was seen in 4 (23.5%) of the cases and one patient died due to rupture of the cyst and anaphylactic shock.

**Conclusion:**

Multi-organ involvement seems to be on the rise in children, this has led to the necessity for physicians to be more aware of clinical features, search, and rule out other organs for involvement diagnosis once a cyst is detected in one organ.

## Introduction

Hydatid disease is a chronic zoonotic helminthic infection caused by larval stage of the dog tapeworm called *Echinococcus granulosus*. Hydatidosis is endemic throughout Middle East and cystic echinococcosis is one of the most important endemic infectious diseases in Iran (1, 2). This disease is usual in areas that cattle, sheep, and dogs are kept. Liver and lungs are common involved organs but in other organs of body such as bones and heart may be found (3–5). In children, lungs are the most common organ infected by larval form of *E. granulosus* (64% in children compared with 20-30% in adults) (6). The parasite commonly transmits to children from direct contact with the feces of dogs (7). Prompt diagnosis, treatment, and surgery may lead to successful control of hydatidosis (3–4). This study was designed to determine pediatric hydatidosis in patients referred to an Iranian referral pediatrics center during 2005-2010.

## Materials and Methods

This study was carried out on the patients diagnosed with hydatidosis among patients referred to the Children Medical Center Hospital in Tehran, Iran, during the 5 years from 2005 to 2010. The center is a teaching hospital of Tehran University of Medical Sciences and is a referral tertiary care center. It admits patients from all over the country with wide range of socioeconomic levels. Demographic data, clinical manifestations, laboratory analysis, type, and site of cyst, and treatment procedures were considered. Radiological data such as computed tomography (CT) of the chest and abdomen, and ultrasonography (USG) records of the files were collected. For eliminating of the cysts, albendazole was prescribed and surgery was applied.

## Results

**Fig. 1:**
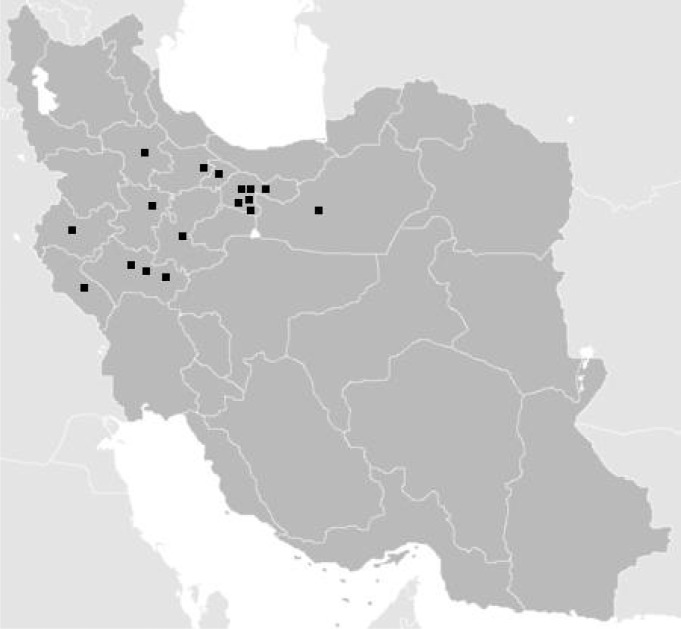
Geographic distribution of the studied hydatid cases in Iran

From end of 2005 to 2010 (5 years), 17 patients with diagnosis of hydatidosis were hospitalized. Nine patients (52.9%) were boys, and 8 patients (47.1%) were girls. The mean age of our patients was 7.8 years and the youngest patient in this study was 4 years. In our study 12 patients (70.6%) of patients (12 out of 17) had records of contact with cattle, sheep or dogs, and 6 patients (35.3%) of patients' parents were farmers. More than half of the patients (10) were from central part of Iran (58.8%). In addition 6 cases (35.3%) and 1 patient (5.9%) were living in the west and northwest of Iran, respectively (Fig. 1).

The percentage of patients with lung and liver hydatid cyst were 41.2% (7 out of 17) and 17.6% (3 out of 17), respectively. Six patients (35.3%) had both of lung and liver cysts, 3 patients (17.6%) had brain cysts (alone or in combination with other organs involvement) and in 2 cases (11.7%) multi-organ involvements (more than 2 organs being involved) were seen (Table 1).

**Table 1:** Site, number, and size of cysts in patients with hydatidosis

Case no.	Lung cysts		Liver cysts		Brain cysts	
	No. of cysts	Diameter of largest cyst (cm)	No. of cysts	Diameter of largest cyst (cm)	No. of cysts	Diameter of largest cyst (cm)
1	1	5.0	0	-	0	-
2	1	3.0	1	5.0	0	-
3	0	-	1	2.0	3	5.0
4	1	3.0	1	4.0	1	5.0
5	0	-	0	-	1	7.0
6	3	4.5	0	-	0	-
7	2	5.6	1	5.0	0	-
8	1	14.0	0	-	0	-
9	1	3.8	1	13.0	0	-
10	0	-	2	2.0	0	-
11	1	6.6	0	-	0	-
12	3	5.0	4	3.0	0	-
13	0	-	1	2.0	0	-
14	1	7.0	0	-	0	-
15	4	10.0	0	-	0	-
16	1	-	0	-	0	-
17	1	5.0	1	10.0	0	-

-Cases 3 and 4 had multiorgan involvement.

-Case 3 had heart cyst (in myocardium) with size of 2.0 cm, peritoneal cyst in size of 3.0 cm and braincysts were in right temporal lobe.

-Case 4 had brain cyst in right temporal/parietal lobe. /-Case 5 had brain cyst in right temporal lobe. / -Case 16 died due to rupture of the cyst and anaphylactic shock.

For 8 patients CT scan was performed and hydatid cysts were approved for all of them (100%). In addition, 11 cases (from 12 patients) had positive results from USG procedure (91.7%). IFA (Immuno Fluorescence Antibody) were requested only for 8 patients and it was positive for 5 cases (63%). Liver and lungs involvement with single cysts were 77.8% (7 out of 9 patients) and 69.2% (9 out of 13 patients), respectively (Table 1). Lungs involvement with multiple cysts happened in 4 patients, that the largest one was 10 cm in diameter. In the liver of 2 patients multiple cysts had grown, that the largest one was 3 cm in diameter (Table 1).

Clinical signs and symptoms of patients with hydatidosis are shown in Table 2. Although the most common symptoms in lung and liver hydatidosis was fever and cough, in cases with brain involvement the major symptom was headache.

**Table 2:** Clinical signs and symptoms in 7 patients with lung hydatidosis, 3 patients with liver hydatidosis, 6 patients with lung& liver hydatidosis & 3 patients with brain hydatidosis

Characteristic	Lung hydatidosis No. of cases (%)	Liver hydatidosis No. of cases (%)	Lung& liver hydatidosis No. of cases (%)	Brain hydatidosis No. of cases* (%)
Cough	6 (85.7)	-	4 (66.7)	-
Fever	5 (71.4)	3 (100)	6 (100)	1 (33.3)
Chest pain	4 (57.1)	-	3 (50.0)	-
Dyspnea	3 (42.9)	-	3(50.0)	-
Hemoptysis	1 (14.3)	-	1 (16.7)	-
Tachypnea	1 (14.3)	-	-	-
Perspiration	-	-	1 (16.7)	-
Hepatomegaly	-	2 (66.7)	4 (66.7)	-
Abdominal pain	-	1 (33.3)	3 (50.0)	-
Anorexia	2 (28.6)	1 (33.3)	2 (33.3)	-
Nausea	1 (14.3)	1 (33.3)	1 (16.7)	1 (33.3)
Vomiting	2 (28.6)	1 (33.3)	2 (33.3)	2 (66.7)
Headache	-	-	-	3 (100)
Convulsion	-	-	-	2 (66.7)

* Brain hydatidosis alone or in combination with other organs involvement

White blood cells (WBC) range in these patients was 6.4 to 26.8×10^3^/µL, with the median value of 13.77×10^3^/µL and neutrophils was the predominant cell type. In addition, leucocytosis was seen in more than half of the patients (10 out of 17). Eosinophilia with 10% or more than 10% was seen in five cases (29.4%) that most of them were cases that had cysts in more than one organ or had multiple cysts in a single organ. Erythrocyte sedimentation rate (ESR) range was 7-104 mm/hr, with average of 54 mm/hr and 58% of cases (7 out of 12) had ESR ≥ 50 mm/hr.

Four cases had recurrence (23.5%) and one patient died due to rupture of the cyst and anaphylactic shock.

## Discussion

Hydatidosis is one of the major endemic infectious diseases in Iran, which is usual in rural zones where cattle, sheep, and dogs are kept (1, 8). In this study youngest patient was 4 years, while in Elburjo and Halezeroglu studies it was 3 and 9 years, respectively (9, 10).

In our study, the finding shows higher incidence of pulmonary hydatid cysts in comparison to hepatic hydatid cysts in children that confirmed other studies (11, 12). It seems that dense tissue of liver and hepatobilitary capsules surrounding it, limit development of cysts, however soft tissue of lungs provides a suitable environment for development of hydatic cysts. In contrast to other studies that incidence of brain hydatidosis is rare (7, 13, 14), in this study brain involvement with hydatid disease occurs in right temporal lobe of 3 cases (17.6%).

CT scan demonstrated hydatid cyst infection in all patients for whom the procedure was carried out. USG techniques are useful for defining most cysts in the abdomen (7), in this study USG procedure was performed for 12 patient and 11cases had positive results in liver or lung (91.7%), so this result show that CT scan and USG can be efficient for diagnosing hydatid cysts.

Most of cysts in lung, liver, and brain were single and six patients (35.3%) had multiple cysts at least in a single organ. Cough, fever, and hepatomegaly were the most common symptoms of pulmonary and hepatic hydatidosis and simultaneous involvement. The prevalence of these symptoms confirms the results of previous studies in Iran and other regions (7, 12, 15, 16).

ESR ≥50 mm/hr was seen in 58% of cases (10/17). These patients had bacterial infections and used antibiotics; thus the increases of ESR can be result of secondary infections. In our study 29.4% (5/17) of patients showed eosinophilia ≥ 10%. Eosinophilia is more often absent in patients with hydatid disease (17) but its level can increase if cysts rupture (18). In addition, an elevated level of eosinophilia can occur in countries where parasitoses is endemic (18).

Comparing our study with previous study in Iran (7), the number of annual cases of hydatidosis in this hospital has increased (31 cases in 10 years against 17 cases in 5 years) and number of multi-organ involvement has raised (3/31 patients (9.7%) against 2 /17 patients (11.8%)). In addition, the number of simultaneous lung and liver involvement has increased (8/31 patients (25.8%) against 6/17 patients (35.3%)). In addition the number of recurrence has ascended (2/31 patients (6%) against 4/17 patients (23.5%)).

In conclusion, multi-organ involvement seems to be on the rise in children, this has led to the necessity for physicians to be more aware of clinical features, search, and rule out other organs involvement once a cyst is detected in any organ. By considering the result of this study, CT scan and USG is recommended for diagnosis of this disease. Also increase in the number of recurrence, presumably show that surgery or treatment has not been with sufficient accuracy thus, we need to investigate new drug that effects on parasites.
